# Supplementary material for: Assessment of Metagenomic Sequencing and qPCR for Detection of Influenza D Virus in Bovine Respiratory Tract Samples
Source: Viruses. 2020 Jul 28;12(8):814. doi: 10.3390/v12080814 (PMC7472010; doi:10.3390/v12080814)
Supplement: Supplementary file 1 [file viruses-12-00814-s001.pdf]

Supplementary Table 1. Summary of viruses detected by Nanopore and MiSeq sequencing.

Sample*	MiSeq	Nanopore (In-house)	Nanopore (WIMP)
6	IDV	IDV	IDV
10	BRSV, BRBV, IDV	BRSV, BRBV, IDV	BRSV, BRBV, IDV
32	BNV	BNV	BNV
50	BRBV, IDV	BRBV, IDV	BRBV, IDV
69	IDV	IDV	IDV
129	BRBV, IDV	BRBV, IDV	BRBV, IDV
170	IDV	IDV	IDV
199	BRSV, IDV, UBPV6	BRSV, IDV, BPIV3	BRSV, IDV, UBPV6
T10	BRSV, IDV	BRSV	BRSV, IDV
13	IDV	IDV	BRSV, BRBV, IDV
T52	BRBV, IDV	IDV	BNV, IDV
42	BCV, IDV	BCV, IDV, BRSV	BCV, IDV, BRSV, BNV
114	BRBV, UTPV1	ND	BRSV, BRBV, IDV, UTPV1
T30	BRBV, IDV	BRBV	BRBV, IDV, BNV
T129	BAV, IDV	BAV, IDV	BAV, IDV, BNV
260	EVE, UTPV1, UBPV6	ND	BRSV, BRBV, IDV
70	BRAV	ND	BRSV, BRBV, IDV
135	BNV	ND	BRSV, BRBV, IDV
T50	ND	ND	BNV, IDV

BCV: bovine coronavirus; IDV: influenza D virus; BRBV: bovine rhinitis B virus; BRAV: bovine rhinitis A virus; BRSV: bovine respiratory syncytial virus; BPIV3: bovine parainfluenza virus 3; EVE: enterovirus E; UTPV1: ungulate tetraparvovirus 1; UBPV6: ungulate bocaparvovirus 6; BNV: bovine nidovirus; BAV: bovine astrovirus; *Samples beginning with T are tracheal, all others are nasal swabs, ND – not detected. WIMP – What's in my pot.
